# Evaluation of the Effect of Ultra-Soft Toothbrushes with Different Commercial Brands on Plaque and Bleeding Indices

**DOI:** 10.30476/DENTJODS.2020.83259.1044

**Published:** 2021-03

**Authors:** Anahita Saffarzadeh, Neda Khodarahmi, Mohammad Mohammadi

**Affiliations:** 1 Dept. of Endodontics, Faculty of Dentistry, Kerman University of Medical Sciences, Kerman, Iran; 2 Periodontist, Kerman, Iran; 3 Dept. of Periodontics, Faculty of Dentistry, Kerman University of Medical Sciences, Kerman, Iran

**Keywords:** Bleeding index, Dental plaque, Toothbrush

## Abstract

**Statement of the Problem:**

Ultra-soft bristles are recommended for individuals with gingival recession, dentinal hypersensitivity, and patients who have undergone periodontal surgeries. However, comparative effectiveness of ultra-soft toothbrushes on dental plaque and bleeding indices has not extensively been studied, and a consensus has yet to be reached on their efficacy.

**Purpose:**

The aim of this study was to investigate the effect of ultra-soft toothbrushes with different commercial brands on plaque and bleeding indices.

**Materials and Method:**

In this crossover randomized clinical trial, 30 participants were selected using convenience sampling method. The subjects were randomly divided into three groups (n=10). In the first session, the bleeding index was recorded. Then each subject was given a toothbrush (Oral B, GUM, or Fuchs), asked to brush at least twice a day using the Bass technique, then avoid brushing for 24 hours after a week and refer for recording the indices. During the second session, bleeding on probing was recorded before brushing, and plaque indices were recorded before and after brushing. Plaque indices before brushing were considered the baseline plaque indices. After one week of washout, each subject used the next toothbrush in terms of the group involved. Turesky plaque index, O’Leary plaque index, and bleeding index were evaluated. The distribution of data was normal. Therefore, ANOVA, t-test, and post hoc tests were used for the analysis of data.

**Results:**

The bleeding and plaque indices decreased significantly compared to the baseline with the use of all the three ultra-soft toothbrushes evaluated (*p*< 0.05), with no significant differences between the three brands (*p*> 0.05) except for the superiority of Fuchs toothbrush in decreasing the Turesky plaque index.

**Conclusion:**

Ultra-soft toothbrushes can reduce plaque index compared to the baseline, but they do not decrease the plaque index up to the optimal level, which might affect their prescription.

## Introduction

Periodontal disease is a prevalent oral condition, which is started by the accumulation of bacterial plaque on tooth surfaces [ 1 ]. Leo *et al*. [ 1 ] reported the importance of plaque on the etiology of gingival inflammation and showed that gingival inflammation will be resolved, and the gingiva will be restored to its normal condition after cleaning the teeth and removal of bacterial plaque. Among all the available means of plaque control, toothbrush is the most common and often the only plaque control means applied by adults and children. The aim of tooth brushing is to remove dental plaque, thereby preventing its evolution into more pathogenic forms, and reducing the risk of dental caries and gingivitis [ 2 - 3 ]. Toothbrushes are different in terms of their size, handle, design, and bristles [ 3 ]. The hardness of toothbrush bristles might affect gingival recession, dentin sensitivity, trauma to soft tissues, and plaque control [ 4 - 7 ]. One of the complications of brushing is the abrasive effect of the brush on the gingiva, which results in gingival recession. Subsequent to gingival recession, there are complications, such as esthetic problems, tooth sensitivity, and greater probability of root surface caries [ 4 ]. Laboratory tests have shown that the direction and number of tooth brushing movements, the force applied, the quality of bristles, and how they are oriented affect the gingival recession associated with tooth brushing [ 8 - 10 ]. 

Since gingival recession results from tooth brushing, many periodontists recommend the use of toothbrushes with medium or soft nylon bristles due to lower epithelial trauma they induce. Khocht *et al*. [ 4 ] showed that individuals using toothbrushes with hard bristles exhibited twice as much gingival recession as those who never used hard toothbrushes, and the rate increased with an increase in the number of times the teeth were brushed. Zimmer *et al*. [ 5 ] showed that toothbrushes with hard bristles removed more bacterial plaque but caused more gingival recession compared to toothbrushes with soft bristles. Considering the prevalence of gingival recession due to tooth brushing and the role of toothbrush bristle hardness, attention has been focused on the use of toothbrushes with softer bristles. 

Recently, toothbrushes with ultra-soft bristles have been commercialized to control plaque in individuals with gingival recession in association with dentin hypersensitivity. The question is to what extent toothbrushes with ultra-soft bristles can remove dental plaque and what effect they might have on gingival indices. Considering the availability of different brands of toothbrushes on the market and lack of information on their quality, it is difficult to select an appropriate toothbrush. Almost 95% of toothbrushes on the market have lower-than-standard bristles, and many have deviated from the standards in terms of the diameter and other dimensions of bristles. In other words, toothbrushes that have been marketed as soft are in fact medium, and those that have been marketed as medium are in fact hard [ 11 ]. 

Limited studies have evaluated the quality of ultra-soft toothbrushes on the market. Moreover, bacterial plaque can produce acidic products, which increase dentin sensitivity, and consequently, failure to eliminate bacterial plaque exacerbates dentin hypersensitivity and affects the gingival and bleeding indices. Therefore, the present study was conducted to evaluate the effect of ultra-soft toothbrushes with different commercial brands on plaque and bleeding indices.

## Materials and Method

In this crossover clinical trial (The Ethics Committee reference number: IR.KMU.REC.1393.531), the study population (n=30) consisted of dental students in the Faculty of Dentistry, Kerman University of Medical Sciences. The sample size was calculated using the following formula:

Z_1_-α /_2_ = 1.96

Z_1_-β = 1.64

δ = 0.56 

σ_1_ = 0.38

σ_1_ = 0.31

n= (Z_1_-α/_2_ + Z_1_-β)^2^(σ_1_^2^+σ_2_^2^) / δ^2^

=> (1.96 + 1.64)^2^(0.38^2^ + 0.31^2^) / 0.56^2^ = 9.93 

Based on the formula above, 10 subjects were included in each group. Opaque, sealed envelopes were used to allocate participants randomly in each group. Considering the crossover design of this study, each toothbrush brand was used by 30 participants, including 18 males and 12 females.

Convenience sampling technique was used to select samples, and the study was carried out in the Department of Periodontics, Faculty of Dentistry, Kerman University of Medical Sciences. The subjects were dental students who were included after signing an informed consent form. 

### nclusion and exclusion criteria

The inclusion criteria consisted of (1) absence of periodontitis and systemic conditions such as type I and type II diabetes mellitus, (2) no use of medications affecting the periodontium, such as nifedipine and cyclosporine, (3) absence of pregnancy, (4) no smoking, (5) no use of anti-inflammatory medications and antibiotics two weeks before the study, (6) no use of partial removable dentures, (7) the presence of at least 18 teeth in the oral cavity, (8) absence of local etiologic factors, such as caries, faulty restorations, and crowns, and (9) presence of Ramfjord teeth, including central incisors and maxillary and mandibular first premolars and first molars. 

All the calculi were removed at least one month before starting the study, and the subjects were instructed to brush their teeth with the Bass technique. The subjects were asked not to use dental floss and mouthwashes during the study period. They were allowed to clean the interdental areas with a toothpick if they had food impaction. 

The subjects were randomly divided into three groups (minimization method) (n=10). First, the subjects in each group were given Oral B, GUM, and Fuchs toothbrushes for 3‒5 days so that they could learn how to brush and how to handle them; in this context, the subjects were asked to brush their teeth at least twice for 2 minutes each time using the Bass technique during the study period. The subject in each group brushed their teeth as following sequence: 

**Group A:** use of GUM toothbrush, wash-out period, use of Fuchs toothbrush, wash-out period, use of Oral B toothbrush

**Group B:** use of Fuchs toothbrush, washout period, use of Oral B toothbrush, washout period, use of GUM toothbrush

**Group C:** use of Oral B toothbrush, washout period, use of GUM toothbrush, washout period, use of Fuchs toothbrush

All the subjects were given anti-cavity Crest toothpaste. During the first examination session, the bleeding index of each subject was recorded. Then, each patient was given the relevant toothbrush and asked to brush their teeth twice a day for 2 minutes each time with the Bass technique for one week, refrain from tooth brushing for 24 hours after one week, and refer for recording the indices. During the second examination visit, the bleeding index [ 12 ] was recorded before brushing, and plaque indices were recorded before and after brushing. The plaque index before brushing was considered as the baseline. All the data were recorded by a clinician blinded to the study procedures. After completion of the use of each toothbrush and recording the indices, there was a one-week wash-out period so that each subject would return to his/her previous plaque control state. During this period, the subjects were allowed to use mouthwashes and dental floss. After this one-week wash-out period, each subject was asked again to use the next toothbrush based on his/her study group. The washout period was one week, and the duration of the use of each toothbrush was one week in all the groups. 

In the present study, the Turesky plaque index, O’Leary plaque index, and bleeding index [ 12 ] were evaluated. Turesky index was used to evaluate the amount of plaque accumulated on Ramfjord index teeth (upper central incisors left upper premolars, right upper first molars, lower central incisors, right lower right first molars and left lower first molars). Each surface of Ramfjord teeth was graded, the total grade of the surfaces was considered as the total grade of each tooth, and the mean of six teeth was reported as the Turesky plaque index of each subject. 

The percentage of the tooth surfaces stained with disclosing tablets in the dentogingival area was reported as the percentage of O’Leary plaque index, which indicated the presence of plaque. 

Gingival bleeding test was carried out by inserting a periodontal probe into the gingival sulcus and moving the probe in a walking manner in a distomesial direction on each tooth. After 60 seconds, the bleeding areas were determined, and the percentage of surfaces with gingival bleeding was calculated. 

### Analysis of data

Kolmogorov-Smirnov test was used to evaluate the normal distribution of data. Since data were distributed normally, ANOVA, t test, and post hoc tests were used for the analysis of data. 

## Results

The results of this study showed that the bleeding index decreased significantly compared to the baseline with the use of all the three ultra-soft toothbrushes (Fuchs, GUM, and Oral B) (*p*< 0.05) (Table 1). There were no significant differences between the three ultra-soft toothbrush brands. The results showed that all the three toothbrushes significantly decreased the percentage of O’Leary plaque index compared to the baseline (*p*< 0.05) (Table 1), with no significant differences between the three toothbrush brands (*p*> 0.05) (Table 2). Although Fuchs toothbrushes resulted in a greater decrease in plaque levels, the differences between the three toothbrushes were not significant (*p*> 0.05).

**Table 1:** Comparison of the average of bleeding index, O’Leary plaque index, and Turesky plaque index before and after tooth brushing with three brands of ultra-soft toothbrushes (GUM, Oral B, and Fuchs)

Indices	Brand of toothbrush	Baseline (Mean±SD)	Post brushing (Mean±SD)	*P* Value
Bleeding Index	GUM	20.26±11.98	12.87±7.74	0.03
	Oral B	20.26±11.98	12.30±7.37	0.01
	Fuchs	20.26±11.98	12.40±7.66	0.02
O’Leary Plaque Index	GUM	75.46±16.15	43.75±18.50	0.00
	Oral B	74.57±19.20	42.94±11.30	0.00
	Fuchs	73.89±20.66	36.85±15.15	0.00
Turesky Plaque Index	GUM	2.08±0.68	0.90±0.48	0.00
	Oral B	2.09±0.66	0.89±0.40	0.00
	Fuchs	1.92±0.77	0.64±0.32	0.00

**Table 2:** Comparison of three brands of ultra-soft toothbrush with each other after intervention in the terms of Bleeding index, O’Leary plaque index and Turesky plaque index

Indices	Comparison Pairs	Mean difference	*p* Value
Bleeding Index	GUM Oral B	0.56	0.99
	Oral B Fuchs	-0.09	1.00
	Fuchs GUM	0.47	0.99
O’Leary Plaque Index	GUM Oral B	0.08	1.00
	Oral B Fuchs	-5.4	0.21
	Fuchs GUM	-5.32	0.29
Turesky Plaque Index	GUM Oral B	0.01	1.00
	Oral B Fuchs	0.25	0.03
	Fuchs GUM	0.26	0.03

The results showed that all the three ultra-soft Fuchs, GUM, and Oral B toothbrushes decreased the Turesky plaque index significantly compared to the baseline (*p*< 0.05) (Table 1). Based on Table 2, there were significant differences in the Turesky plaque index after intervention between Oral B and Fuchs and between GUM and Fuchs toothbrushes (*p*< 0.05). Post hoc tests showed the greatest difference between GUM and Fuchs toothbrushes (Table 2).

## Discussion

The mechanical plaque control has a critical role in the prevention and treatment of periodontal diseases [ 1 ]. The presence of bacterial plaque results in the initiation and progression of periodontal diseases and dental caries [ 3 ], interfering with the healing of periodontal surgery wounds [ 13 ]. Moreover, the best way to eliminate this dental plaque is to use a toothbrush [ 3 ]; hence, it is necessary to use manual toothbrushes with proper hardness of bristles to eliminate a high percentage of bacterial plaque. Although several studies have evaluated the efficacy of different toothbrushes in eliminating plaque, there is still controversy over the superiority of toothbrushes over each other [ 14 - 16 ]. However, ideally, a toothbrush should have the capacity to clean the tooth surfaces of microbial plaque with the least side effects. Toothbrushing might be associated with some complications, such as gingival recession due to traumatic brushing, abrasion of restorations, especially in cervical areas, abrasion of the tooth protective layers, and gingival traumas [ 5 , 17 - 18 ]. 

Several factors, including the hardness of toothbrush bristles, have a role in the incidence of untoward complications [ 6 - 7 ]. In clinical studies carried out on independent groups, confounding factors, such as individual differences, could affect the results of the study. Therefore, the crossover pattern was used in the present study to decrease the effects of confounding factors as far as possible. The results of the present study showed that the use of Fuchs, Oral B, and GUM ultra-soft toothbrushes did not result in significant differences in decreases in O’Leary plaque index and gingival bleeding index (*p*> 0.05); however, Fuchs toothbrush decreased Turesky plaque index significantly (*p*< 0.05). 

In the present study, the O’Leary plaque index decreased to 43%, 36%, and 42% with the use of ultra-soft GUM, Fuchs, and Oral B toothbrushes, respectively, indicating significant decreases in plaque compared to the baseline. However, an important consideration in this respect is the failure to decrease the O’Leary plaque index below 20% [ 19 ], which is one of the criteria for the evaluation of oral health. 

Parizi *et al*. [ 20 ] compared an electric toothbrush with two manual toothbrushes using the O’Leary plaque index. The results showed no statistically significant difference concerning plaque control between Jordan Power electric toothbrush and either of Oral-B Advantage or Panbehriz Classic manual toothbrushes after two weeks.

Zimmer *et al*. [ 5 ] evaluated the effect of the extent of plaque control in manual toothbrushes with a different hardness of toothbrush bristles. The results of the study showed better plaque control with the use of toothbrushes with medium and hard bristles compared to those with soft bristles. Ultra-soft toothbrushes were not evaluated, and therefore, the results of that study cannot be compared with those of the present study. 

Based on a report by Niemi *et al*. [ 21 ], although toothbrushes with hard bristles can effectively remove the microbial plaque from the tooth surfaces, they simultaneously exert detrimental effects on the gingival tissue. A study by Carvalho *et al*. [ 17 ] confirmed an increase in traumas to gingival tissues with the use of toothbrushes with harder bristles. 

In recent decades, several new toothbrush designs have been introduced, and manufacturers have made efforts to improve their efficacy and safety [ 22 ]. Given the discomfort and sensitivity of periodontal surgical sites, dentin hypersensitivity, and gingival recession, attention has been focused on manufacturing toothbrushes with thin and very soft bristles. In this context, ultra-soft toothbrushes have been designed, manufactured, and marketed by various companies [ 7 , 13 , 17 ]. 

Since toothbrushes with different designs can result in different degrees of plaque control [ 23 ], it is necessary to evaluate ultra-soft toothbrushes in terms of different levels of plaque removal as a need for selecting toothbrushes by patients. 

Several studies have evaluated ultra-soft toothbrushes and have reported different results. Vowels and Wade [ 24 ], compared a toothbrush with bristles measuring 0.28 mm in diameter with a toothbrush with a bristle diameter of 0.13 mm. The results showed that the toothbrush with 0.28-mm bristles was significantly better in controlling plaque. Based on the results of this study, a decrease in the diameter of toothbrush bristles can be a factor affecting the amount of plaque control, consistent with the results of the present study, that is, ultra-soft toothbrushes decrease the amount of plaque, but this decrease is not sufficient. 

In another study by Beatty *et al*. [ 25 ], no significant differences were detected between toothbrushes with bristles measuring 0.2 and 0.18 mm in diameter, which might be attributed to the minor differences in the diameters of the bristles. Hedge *et al*. [ 26 ] compared a Thermoseal ultra-soft toothbrush (0.18 mm of bristle diameter) with a Plakoff soft toothbrush in terms of the amount of plaque removed. They employed Turesky plaque index for comparison, which increased over time with the use of the ultra-soft toothbrush, increasing from 2.93 at baseline to 3.25 after 14 days. However, with the use of the soft toothbrush, the plaque index decreased from 3.17 to 2.59, which was significant statistically [ 26 ].

In the present study, the Turesky plaque index decreased to 0.64, 0.90, and 0.89 with the use of Fuchs, GUM, and Oral B toothbrushes, respectively, after one week, which was significant compared to baseline. Compared to the results reported by Hedge *et al*.[ 26 ], the amount of plaque in the current study exhibited a decreasing trend, while in their study , an increase was observed in the amount of plaque with the use of an ultra-soft toothbrush. This difference might be attributed to the features in toothbrush design, including the number of tufts, the number of bristles in each tuft, and so on. 

Motevecci *et al*. [ 27 ] evaluated the effect of plaque control with the use of Meriodl-Perio ultra-soft toothbrush with a standard soft toothbrush after periodontal surgery .They reported that O’Leary plaque index reached 23% and 32%, respectively, after one month with the use of ultra-soft and soft toothbrushes, indicating significantly greater effect of ultra-soft toothbrush. In that study, the conical shape of the bristles of Meridol-Perio toothbrush was reported as the superiority of this toothbrush in removing the bacterial plaque. In the present study, the O’Leary plaque index reached 36%, 43%, and 42% after one week, with the use of Fuchs, GUM and Oral B toothbrushes, respectively, which were significant compared to the baseline values; however, the plaque index was higher compared to the study by Motevecci *et al*. [ 27 ], which might be attributed to differences in toothbrush designs. 

Based on the results of the present study and the limited number of studies carried out on the characteristics of ultra-soft toothbrushes from a clinical viewpoint, it might be concluded that these toothbrushes can have a role in decreasing plaque compared to baseline, but the amount of plaque does not decrease optimally.

Since hardness and elasticity of toothbrush bristles decrease over time, we suggest that the use of ultra-soft toothbrushes should be confined to short durations, such as their use after periodontal surgeries or dentin hypersensitivity cases resulting from gingival recession. In relation to the prescription of an appropriate toothbrush, each individual’s ability to brush can be a determining factor. For example, it is recommended that individuals with poor oral hygiene use toothbrushes with medium bristles; the use of ultra-soft and even soft toothbrushes will not adequately improve plaque control in such individuals. One limitation of the present study was sampling method and use of dental students as samples. These facts reduce the generalizability of the results to the community and further studies with different designs are needed.

## Conclusion

Based on the results of the present study, there were no significant differences in O’Leary plaque index and the decrease in gingival bleeding with the use of different brands of ultra-soft toothbrushes, except for the superiority of Fuchs toothbrush in decreasing Turesky plaque index. All the toothbrushes improved the plaque index and gingival bleeding compared to the baseline, but the amount of plaque did not decrease optimally, and this can affect the administration of these toothbrushes.
